# Neurophysiological Insights into the Pathophysiology of Stiff‐Person Spectrum Disorders

**DOI:** 10.1002/mdc3.14328

**Published:** 2025-01-08

**Authors:** João Moura, Lorenzo Rocchi, Michael Zandi, Bettina Balint, Kailash P. Bhatia, Anna Latorre

**Affiliations:** ^1^ Department of Neurology Unidade Local de Saúde de Santo António Porto Portugal; ^2^ ICBAS School of Medicine and Biomedical Sciences University of Porto Porto Portugal; ^3^ Unit of Multidisciplinary Research in Biomedicine (UMIB), ICBAS University of Porto Porto Portugal; ^4^ Department of Medical Sciences and Public Health University of Cagliari Cagliari Italy; ^5^ Department of Neuroinflammation UCL Queen Square Institute of Neurology London United Kingdom; ^6^ Department of Neurology Zürich University Hospital Zurich and University of Zurich Switzerland; ^7^ Department of Clinical and Movement Neurosciences UCL Queen Square Institute of Neurology London United Kingdom

**Keywords:** stiff‐person spectrum disorders, neurophysiology, transcranial magnetic stimulation, continuous motor unit activity, exteroceptive reflex, startle

## Abstract

**Background:**

Stiff Person Spectrum Disorders (SPSD) are classically defined by the presence of muscle stiffness, spasms and hyperactivity of the central nervous system. There is a notable correlation between neurophysiological features and the clinical hallmark of SPSD, which has greatly encouraged the use of these techniques for diagnostic purposes. Besides, electrophysiological techniques allow for a functional evaluation of the ‘hyperactivity of the CNS’, thus offering the opportunity to clarify the mechanisms underlying this disorder. This review delves into the current knowledge on the electrophysiological aspects of SPSD, highlighting the pivotal role of various studies in unravelling its pathophysiology.

**Methods:**

Literature review for studies on SPSD that included a neurophysiological evaluation.

**Results:**

We first examined the abnormal neurophysiological findings of SPSD across the central nervous system, from the spinal circuit to the motor cortex. Subsequently, we discussed their pathological implications and explored how these findings can be interpreted within the framework of an immune‐mediated disorder.

**Conclusions:**

Two primary questions remain unanswered: the localization of the primary abnormality within the central nervous system and the connection between the autoimmune basis of SPSD and its neurophysiological aspects. Addressing these questions could provide invaluable insights into SPSD etiology and targeted therapeutic strategies.

Stiff‐person spectrum disorders (SPSD) comprise a group of rare neurological conditions classically defined by the presence of muscle stiffness, spasms, and hyperactivity of the central nervous system (CNS).^1^ Muscle stiffness is progressive, predominantly affecting the axial muscles, and associated with spasms that can be spontaneous or triggered by external sensory stimuli or emotional stress^2^; the former often elicits an exaggerated startle response, which is another core feature of SPSD. SPSD represents a continuum ranging from the stiff‐limb syndrome, where symptoms are confined to only 1 limb, to the classical stiff‐person syndrome, mainly presenting with axial involvement, to progressive encephalomyelitis with rigidity and myoclonus (PERM), which exhibits axial and limb rigidity, with prominent myoclonus, dysautonomia, and a more aggressive course.^3^, ^4^, ^5^

Most SPSD cases (60%–80%) have autoantibodies against glutamic acid decarboxylase (GAD), a rate‐limiting enzyme responsible for the synthesis of the inhibitory neurotransmitter GABA in the CNS.^6^, ^7^ Other antibodies targeting gephyrin and amphiphysin are mainly found in paraneoplastic variants of SPSD, whereas PERM is commonly associated with antibodies against glycine receptors (GlyR‐Abs).^8^, ^9^ The diagnosis of SPSD is challenging due to its rarity and clinical heterogeneity, and relies on the integration of clinical, serological, and cerebrospinal fluid analyses.^2^, ^7^ However, especially in the past, when antibodies were not widely available or in seronegative patients, electrophysiological studies were viewed as relevant for the diagnosis of SPSD.^2^, ^10^, ^11^, ^12^ These complement the clinical examination and provide objective information on the symptoms observed; they consist of techniques used to investigate the motor unit activity of the stiff muscles, and exteroceptive reflex and startle reflex (SR). Additionally, electrophysiological techniques allow for a functional evaluation of the “hyperactivity of the CNS,” namely CNS increased excitability (or reduced inhibition), thus providing the opportunity to determine the mechanisms underlying the SPSD.

This narrative review delves into the current state of knowledge of the electrophysiological aspects of SPSD, highlighting the pivotal role of various studies in unraveling its pathophysiology. To do so, we reviewed the literature for studies on SPSD that included a neurophysiological evaluation. Because misdiagnosis is common in SPSD, we assessed if the included studies fulfilled the diagnostic criteria proposed by Chia and coworkers.^13^ These results are presented in Table S1. All the included studies were based on patients with a clinical syndrome compatible with SPSD and in which alternative diagnoses were excluded.

## Continuous Motor Unit Activity and Spinal Cord Reflexes

The clinical hallmark of SPSD is progressive stiffness predominantly involving the axial and proximal muscles, with superimposed spasms. The electrophysiological correlate of stiffness is the continuous motor unit activity (CMUA) at rest, which characterizes SPSD but is indistinguishable from voluntary muscle activation. Initial descriptions of the electromyogram in SPSD refer to a steady low‐frequency firing of normal motor units with fluctuating intensity and location, mostly involving the trunk and proximal upper‐ and lower‐limb muscles.^1^, ^14^ This constant firing is present even at rest, is not altered by relaxation attempts, and reflects persistent tonic contraction.^11^, ^15^, ^16^, ^17^ The motor unit potentials in SPSD have normal configuration and firing rates^1^, ^12^; denervation potentials (fibrillations or positive wave discharges), complex repetitive discharges, myokymia, or myotonic potentials are absent.^18^ This implies that, from an electrophysiological perspective, there is preservation of inputs to motor units, without signs of muscle denervation. Although not specific, CMUA is the most consistently used electrophysiological feature to define SPSD in the literature^19^, ^20^, ^21^, ^22^, ^23^, ^24^; interestingly, the same electrodiagnostic marker has been replicated in other species, namely in stiff dog^25^ and stiff horse.^26^ Currently, there are no robust data on the prevalence of CMUA in different muscle groups and respective diagnostic accuracy in SPSD.

Relevant physiological information can be obtained when considering the relation between agonist and antagonist muscles, such as their simultaneous activation, a phenomenon known as co‐contraction, that is particularly evident when assessing the SPSD patient in a supine position. In normal physiology, spinal inhibitory interneurons coordinate the activity of agonist and antagonist muscles by reciprocal inhibition.^27^, ^28^ In SPSD, however, CMUA persists during the contraction of antagonist muscles, indicating a failure of reciprocal inhibition mechanism or other related spinal circuits.^12^, ^29^ This finding suggests the dysfunction of a normal physiologic GABAergic inhibitory mechanism, leading to continuous firing of γ motor neurons due to lack of inhibitory signaling and resulting in overstimulation of muscle spindles, clinically expressed as stiffness.^30^ Therefore, the co‐contraction of agonist/antagonist points toward GABAergic dysfunction being a central aspect of SPSD.

The simplest spinal cord reflex is the H‐reflex, which is at least partly considered the neurophysiological correlate of the tendon reflex and reflects the activation of spinal motor neurons by an afferent volley from group Ia fibers.^31^ The H‐reflex can be used as a probe to study spinal cord circuits related to different physiological properties. The most commonly tested are (1) Ia afferent inhibition (ie, control of sensory feedback at presynaptic inhibitory synapses of afferent terminals on spinal motor neurons, mediated by GABAergic interneurons); (2) reciprocal inhibition (ie, the inhibition of the spinal motor neurons, through Ia GABAergic interneurons, evoked by contraction of antagonist muscles)^28^, ^32^; (3) nonreciprocal Ib inhibition, mediated by the activation of Golgi tendon organs and following Ib pathways^33^; and (4) recurrent inhibition (ie, inhibition of spinal motor neurons by Renshaw cells, through the co‐release of GABA and glycine, which are activated by axons from the same motor neurons).^34^ These have been only seldom investigated in SPSD. In the pivotal study by Floeter and colleagues,^35^ the most consistent finding was the loss of vibration‐induced H‐reflex inhibition, suggesting reduced presynaptic inhibition, mediated by GABAergic interneurons, in these patients.^11^, ^36^ However, the presynaptic component of the reciprocal inhibition, also mediated by GABAergic neurotransmission, was preserved,^35^, ^37^ a finding that seems to be in contrast with the previous one. The nature of this discrepancy is not clear, and it was attributed by the authors to a variable impairment of GABAergic transmission within the subjects, as well as possible pathophysiological heterogeneity.^35^ In the same study, recurrent inhibition was lower than in healthy controls, although the difference was not statistically significant^35^; this suggests a partial preservation of postsynaptic inhibition mediated by glycine transmission. Finally, it has been shown that physiological nonreciprocal Ib inhibition obtained by stimulation of afferents from the gastrocnemius medialis^33^ is replaced by facilitation in SPSD.^36^

Overall, these findings suggest that in SPSD there is a predominant dysfunction of presynaptic inhibitory mechanisms, which are mainly mediated by GABAergic interneurons.^38^ Other forms of spinal cord inhibition, such as recurrent and (in part) reciprocal inhibition, which may depend also on glycinergic transmission,^39^ seems more preserved. These findings may be explained by the observation that anti‐GAD antibodies are much more common than anti‐glycine in SPSD. However, a serological confirmation of this hypothesis has yet to be provided.

## Exteroceptive Reflexes

Exteroceptive reflexes are generally defined as polysynaptic muscle responses elicited by different sensory modalities (tactile, electrical, auditory, or visual).^4^, ^10^ More specifically, cutaneomuscular reflexes are a form of exteroceptive reflexes induced by electrical stimulation of peripheral sensory or mixed nerves, which are considered a hallmark feature of SPSD,^11^, ^16^, ^17^ despite the lack of evidence of their absence in other forms of hypertonia. To elicit a cutaneomuscular reflex, electrical stimuli are delivered at intensities above the somatosensory threshold, usually in train of four or five pulses, and motor responses are recorded from distal upper‐ and lower‐limb muscles, as well as axial muscles.^18^ In SPSD, these responses are enhanced, habituate poorly, have abnormally short latencies, and spread into muscles normally not involved in the reflex.^16^, ^29^ The clinical counterpart of exaggerated cutaneoumuscular reflexes is probably represented by the stimulus‐induced muscle spasms that characterize SPSD. The term “spasmodic reflex myoclonus” has been suggested to describe a violent, jerky, and stereotypical response to electrical stimulation.^11^, ^18^, ^29^ This has been observed in SPSD mainly as lower‐trunk (both ventral and dorsal) muscle jerks induced by stimulation of the tibial nerve,^5^, ^11^ with a characteristic electromyography (EMG) pattern, that is, 1 to 3 bursts occurring at latencies of 70, 120, and 170 ms with symmetrical and simultaneous activation of the antagonist muscles.^11^

Other external stimuli may lead to exaggerated muscle responses in SPSD. For instance, unexpected acoustic stimuli may elicit a response similar to that described earlier, with EMG recordings showing bilateral activation of upper‐ and lower‐limb muscles^29^, ^40^; however, this might be considered a form of SR, which is described later. In SPSD, exteroceptive reflexes obtained with mechanical and electrical (either single shock or pulse trains) stimulation exhibit less habituation compared to healthy subjects.^5^, ^11^, ^16^, ^17^, ^19^, ^41^ Importantly, the motor conduction time and evoked potentials induced by electrical stimulation of the limbs appear to be preserved in SPSD.^5^, ^11^

The mechanisms underlying exaggerated exteroceptive reflexes in SPSD have not yet been fully elucidated; similar to what occurs with the augmentation of the blink reflex (BR) and other exteroceptive brainstem reflexes (as discussed later), the main hypotheses include abnormal transmission of afferent impulses to spinal motor neurons or abnormal descending motor control.

### Startle Reflex

The SR is a physiological response to unexpected stimuli, mainly auditory, that elicits a stereotyped rostral‐caudal muscle recruitment (early blink response followed by sternocleidomastoid activation and then masseter, trunk, and limb muscles), with a pattern and latency compatible with an origin in the lower brainstem.^42^ Patients with SPSD experience an exaggerated SR^43^, ^44^ characterized by increased amplitude, enhanced gain of reflex transmission, hypersynchronous response in the trapezius and sternocleidomastoid followed by prolonged spasms in the same and other muscles, and poor habituation.^45^ In EMG recording, these features are reflected by the shorter‐onset latencies of EMG responses, longer burst durations, reduced habituation to repeated stimuli, and more consistent activation of limbs and lumbar spinal muscles.^40^, ^46^

A specific form of SR normally suppressed in healthy subjects is the head retraction reflex (HRR), which consists of a vestigial withdrawal reflex of the head in response to glabellar tapping or electrical stimulation of the supraorbital nerve.^47^ The HRR is a characteristic feature of patients with SPSD and hereditary hyperekplexia. Electrophysiological findings show that it is characterized by 2 excitatory components (R1, presumably oligosynaptic; and R2, presumably polysynaptic and of variable latency) separated by a silent period, with muscle activity spreading subsequently from the trapezius to the sternocleidomastoid, thoracic, and, sometimes, paraspinal muscles.^48^ Although the HHR clinically resembles a mild SR, it is considered a type of exteroceptive reflex.

### BR Recovery Cycle

The BR is produced by a polysynaptic brainstem circuitry. It is elicited by electrically stimulating the supraorbital branch of the trigeminal nerve on 1 side, resulting in a reflexive activation of the facial nucleus, causing bilateral contraction of the orbicularis oculi muscle.^49^ The response is characterized by a first ipsilateral component (R1) that reflects the activation of an oligosynaptic pathway in the pons, followed by a bilateral polysynaptic component (R2), mediated by interneurons in the medulla.^50^, ^51^ Recovery curves of both R1 and R2 can be obtained by applying a double‐pulse technique to the supraorbital nerve at varying interstimulus intervals (ISI).^50^, ^52^ Because of after‐hyperpolarization potentials and/or negative feedback circuits, the reflex response is depressed after a first afferent volley; therefore, the excitability of the reflex can be assessed by measuring the size of the response to a second stimulus (test) delivered at progressively longer intervals after the initial (conditioning) stimulus.^53^ SPSD is associated with a shortening of the R2 recovery time after conditioning, which likely reflects enhanced excitability of brainstem interneuronal circuitry.^45^, ^54^ In cases where there is unilateral involvement of the face by stiffness in SPSD, an asymmetric BR with increased amplitude of the ipsilateral R1 and R2 components may be observed.^55^

Excessive startle responses as seen in SPSD and hereditary hyperekplexia are thought to reflect defective inhibition due to impaired glycinergic transmission.^56^ The combination of excessive SR and disinhibition of other brainstem reflexes, such as the BR, suggesting an exaggerated brainstem reflex excitation and attenuation of reflex inhibition, implies a defective inhibition mediated by GABAergic and glycinergic brainstem interneurons in SPSD.^45^ It remains unresolved whether the primary abnormality lies in the brainstem or is caused by impaired descending modulation of inhibitory circuits by descending pathways.

## Transcranial Magnetic Stimulation

Transcranial magnetic stimulation (TMS) offers a noninvasive means of assessing net motor cortex (M1) excitability, as well as inhibitory and facilitatory intracortical circuitries in healthy subjects and patients with various nervous system diseases, including movement disorders.^57^, ^58^ Various TMS paradigms are employed for this purpose, divided into single‐pulse TMS, where the primary outcome measure is the motor evoked potential (MEP), and paired‐pulse (pp) TMS, involving the delivery of 2 stimuli (a conditioning stimulus and a test stimulus) through the same coil, with a specific ISI. After pp‐TMS, the degree of inhibition or facilitation of the test MEP depends on the ISI and the intensity of the conditioning pulse, thereby enabling the investigation of intracortical inhibitory and facilitatory circuits.^59^, ^60^, ^61^ A first study by Sandbrink and colleagues^62^ investigated 8 SPSD patients with single‐pulse and pp‐TMS paradigms, recording MEPs from upper and lower limbs. The authors found that SPSD patients had normal central motor conduction velocities, motor threshold for MEP in the legs, and stimulus/response recruitment curves in hands and legs; this suggests intact transmission through the corticospinal tract and normal M1 net excitability. However, SPSD patients had shorter cortical silent period (CSP) after TMS targeting the leg area of M1. The CSP is a period of EMG silence after an MEP obtained during tonic contraction of the target muscle and is believed to reflect GABAB‐mediated inhibition^63^, ^64^; therefore, this finding supports cortical inhibition deficit in SPSD.^62^ Furthermore, when using pp‐TMS to assess intracortical excitability by means of short‐interval intracortical inhibition (SICI) and intracortical facilitation (ICF), the same authors found reduced SICI and increased ICF measured in hand muscles. These stimuli did not influence the H‐reflex, indicating that spinal excitability was not implicated in the findings mentioned earlier. Reduced MEP thresholds and absent CSP have been reported in 1 case,^65^ whereas another study, using the aforementioned single‐pulse and pp‐TMS paradigms, found enhanced ICF and reduced SICI and CSP in SPSD compared to controls.^66^ Interestingly, in a further study, the authors found that GABAergic medications significantly reduced ICF but did not affect SICI in patients; moreover, M1 excitability was increased to a larger extent in patients with GAD antibodies compared to patients without, and cortical excitability was positively correlated with GAD antibody levels in the cerebrospinal fluid.^66^ This is a relevant finding, as it is known that SICI, which is likely mediated by GABAA neurotransmission,^67^, ^68^ is enhanced by GABAergic drugs, whereas ICF, which may be mediated by cortical glutamatergic synapses, is suppressed by them.^68^ According to the authors, this evidence suggests that the GABAergic medication partly compensated for M1 hyperexcitability in SPSD patients. Similarly, in a single case, SICI was unchanged by immunotherapy (intravenous immunoglobulin, mycophenolate mofetil, and prednisone), whereas ICF dramatically decreased after it, together with a reduction of GAD antibodies.^69^ That said, it is important to approach the conclusions drawn from TMS studies with caution, given the small number of patients involved.

## Discussion

Neurophysiological studies provide valuable insight into CNS changes in SPSD, elucidating its pathophysiological mechanisms (Table 1). The correlation between neurophysiological features and the clinical hallmark of SPSD can be striking: whereas CMUA manifests clinically as stiffness, the exaggerated reflex responses result in the characteristic muscle spasms. However, for other measures, the clinical correlation is less apparent, although speculation regarding their significance can be ventured.

**TABLE 1:** Summary of electrophysiological findings in SPSD and possible underlying pathophysiological mechanisms

Neurophysiological technique	Main findings	Clinical counterpart	Possible pathophysiological implications
EMG	CMUA—steady low‐frequency firing of normal motor units at rest, mostly axial/proximal limbs Normal potentials and absent denervation	Stiffness	Persistent muscle activity due to failure of reciprocal inhibition or other related spinal inhibitory mechanisms
Spinal inhibitory circuit	Reduced presynaptic inhibition (loss of vibration‐induced H‐reflex inhibition) Normal presynaptic component of reciprocal inhibition Normal recurrent inhibition Abnormal nonreciprocal Ib inhibition (Ib soleus H reflex inhibition replaced by facilitation)	Muscle spasms/stiffness/startle/“spasmodic reflex myoclonus”	Dysfunction of (GABAergic) presynaptic inhibition (with GAD antibodies being present in 70% of cases) Recurrent (glycinergic) postsynaptic inhibition seems to be preserved (anti‐glycin is found only in 10%–20% of SPSD cases)
Exteroceptive reflexes	Mechanical, electrical, and acoustic stimuli induce responses that are enhanced, habituate poorly, have short latencies, and spread to other muscles HRR SR: exaggerated, with hypersynchronous responses and poor habituation BRRC: shortening of the R2 recovery time after conditioning		Abnormal transmission of afferent impulses to spinal motor neurons Abnormal descending motor control Decreased (glycinergic) inhibition of brainstem interneuronal circuits
TMS	Normal transmission through the corticospinal tract and M1 net excitability Shorter/absent cortical silent period Reduced SICI Increased ICF GABergic stimulation reduces ICF but does not affect SICI Cortical excitability is positively correlated with anti‐GAD titer in the cerebrospinal fluid	Muscle spasms/stiffness?	Reduced intracortical inhibition/increased facilitation (mediated by GABA and glutamate, respectively)

Abbreviations: SPSD, stiff‐person spectrum disorders; EMG, electromyography; CMUA, continuous motor unit activity; HRR, head retraction reflex; SR, startle reflex; BRRC, blink reflex recovery cycle; TMS, transcranial magnetic stimulation; SICI, short‐interval intracortical inhibition; ICF, intracortical facilitation; GAD, glutamic acid decarboxylase.

The CMUA found in SPSD does not seem to stem from alterations in peripheral nerves or spinal motor neurons, which have normal excitability.^12^ On the contrary, a failure of the normal reciprocal inhibition points to a dysfunction of spinal inhibitory interneurons, and in particular of mechanisms mediating presynaptic inhibition of Ia terminals. However, the pattern of involvement of spinal inhibitory reflexes is variable, because some presumptive GABAergic or glycinergic synapses seem to be preserved, whereas others do not. These findings could be explained by previously unrecognized GABAergic contributions to presumed glycinergic reflexes or even by differences in susceptibilities intrinsic to specific neural populations.^35^ Another hypothesis is that the primary deficit lies in supraspinal structures, which fail to control spinal circuits.^70^ In this regard, it has been proposed that impairment of GABAergic neurotransmission in the motor pathways leads to unopposed activation of spinal γ motor neurons, resulting in motor unit firing of agonist and antagonist muscles at rest.^30^, ^71^, ^72^ However, if this were true, the gain of spinal monosynaptic reflexes would likely be increased, but this has not been observed.^12^

The hypothesis of a central origin for the impairment of motor unit inhibition is supported by studies showing that centrally acting GABAergic drugs such as benzodiazepines and baclofen suppress CMUA.^29^, ^73^ These compounds, however, act on both brain and spinal cord receptors; therefore, understanding the site of action linked to clinical improvement can be challenging. Moreover, studies on the exteroceptive reflex and SR show that the dysfunction of inhibitory connections extends beyond spinal interneuronal networks to involve descending fibers.^45^, ^54^ For instance, enhanced polysynaptic brainstem reflexes are often presumed to be released by the lack of suppression provided by descending fibers from the cortex. This is the hypothesized mechanism for the decreased recovery of the R2 component of BR,^54^ which has been described in several movement disorders involving basal ganglia dysfunction and thought to be caused by reduced inhibitory drive from the basal ganglia and M1 to brainstem circuits.^52^, ^74^ Finally, cortical involvement in SPSD is confirmed by TMS studies, which showed reduced inhibition and increased facilitation of the M1, supporting the premise of a supraspinal dysfunction of inhibitory mechanisms.^62^ Similarly, the dysfunction in interneurons in the upper cortical layers mediating GABAergic presynaptic inhibition onto pyramidal neurons also increases cortical hyperexcitability in SPSD.^75^

**FIG. 1:**
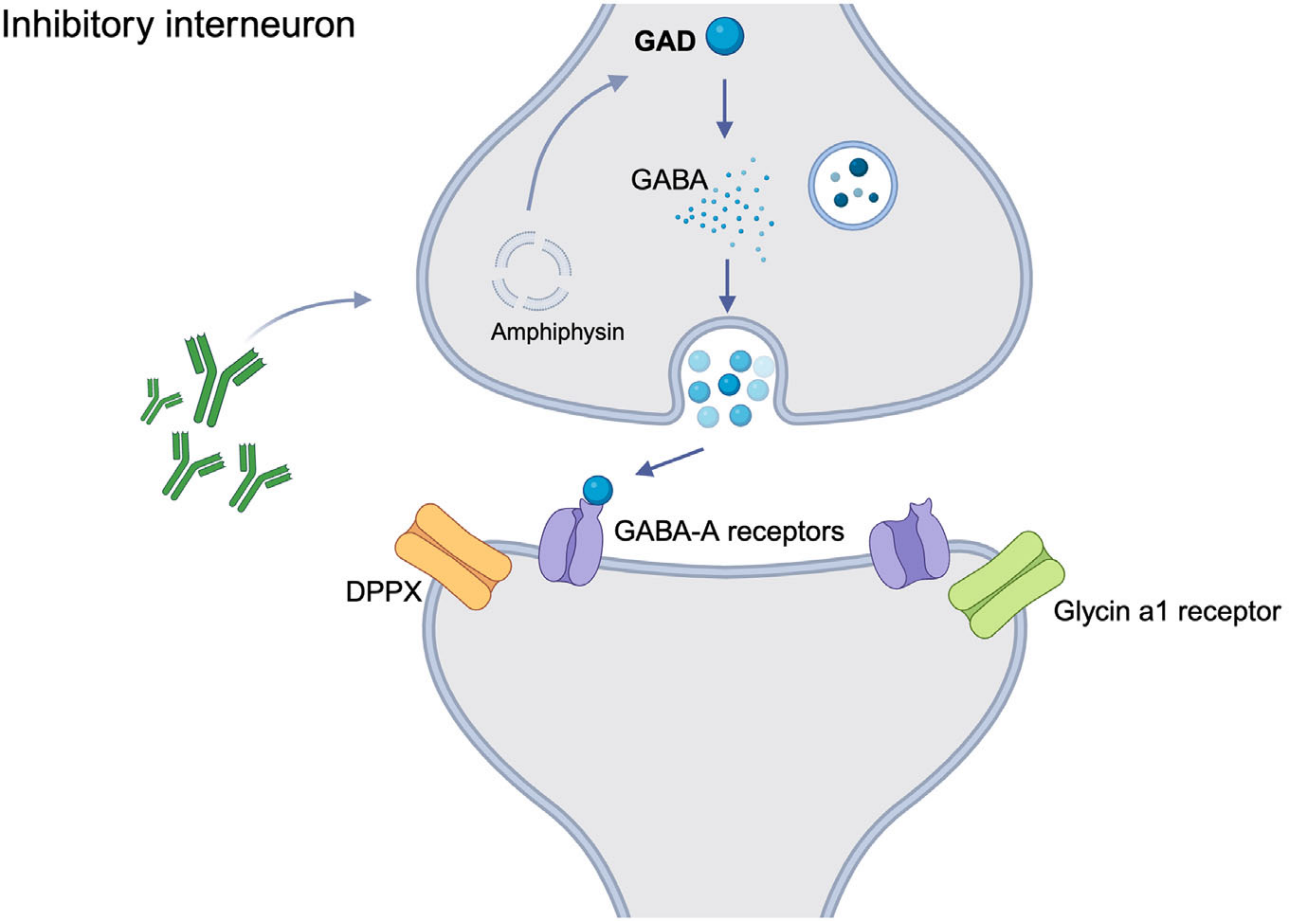
Diagrammatic representation of a GABAergic inhibitory synapse. The figure shows some of the intracellular targets of known antibodies associated with SPSD (stiff‐person spectrum disorders) (GAD [glutamic acid decarboxylase], amphiphysin). Extracellular targets of known antibodies (DPPX [dipeptidyl peptidase‐like protein], glycine receptor) are represented in the postsynaptic membrane.

Overall, these studies suggest the presence of an inhibitory dysfunction in SPSD that extends from cortical networks to the spinal cord; it remains to be elucidated, however, if such alterations in the brainstem or spinal cord circuitry are primary or if they are driven by aberrant drive from the cortex. Additionally, the connection between the autoimmune basis of SPSD and its neurophysiological aspects remains unresolved. Although GABA synthesis is inhibited in vitro by the purified anti‐GAD IgG fraction derived from SPSD patients^76^ and SPSD patients with high anti‐GAD CSF titers have reduced GABA levels in the CSF^72^ and brain,^77^ the intracellular location of the target enzyme puts a direct pathogenic role of anti‐GAD antibodies into question.^78^ Figure 1 shows a GABAergic synapse for reference. Furthermore, from a neurophysiological perspective, there is no evidence demonstrating that anti‐GAD antibodies impair normal inhibitory mechanisms in animal models.^30^ This observation seems to contradict the consistent finding of GABAergic involvement in the spinal and cortical inhibitory circuitry in SPSD, as indicated by spinal cord reflexes and TMS studies, and raises the question if anti‐GAD antibodies are the primary causes of these abnormalities or if other mechanisms are involved. In fact, although GABA plays a role in this context, these studies have shown contrasting results regarding its implication. For instance, GABAergic presynaptic inhibition is not always impaired, and SICI is not restored or altered by GABAergic medications and immunotherapy in SPSD. This has important treatment implications because GABA mimetics are the most common symptomatic drugs used in this condition.^79^ Apart from anti‐GAD, SPSD can present with other antibodies targeting antigens associated with inhibitory synapses, such as amphiphysin, glycine‐receptor (GlyR), gephyrin, dipeptidyl peptidase‐like protein (DPPX), and GABA‐A receptor‐associated protein.^30^ The pathogenic role of the antibodies targeting GlyR is clearly explained, considering the extracellular location of the target epitopes.^80^ With glycine being a known inhibitory neurotransmitter, these antibodies result in dysfunctional disinhibition of neural circuits,^81^, ^82^ confirmed by abnormal neurophysiological patterns such as excessive SR and prolonged BR recovery cycle.^30^ DPPX is an extracellular regulatory protein of the Kv4.2 potassium channels, which has an inhibitory function relevant to the attenuation of back‐propagation of action potentials in the nervous system.^83^ Anti‐DPPX IgG disrupts this inhibitory function, likely resulting in hyperexcitability of the structures involved. The widespread distribution of the Kv4.2 potassium channels results in a diverse phenotype that is not circumscribed to PERM or SPSD.^30^ All the other recognized target antigens are located intracellularly. This includes the paraneoplastic form of SPSD, which is commonly associated with antibodies directed against amphiphysin. This nonintrinsic membrane protein co‐localizes with GAD in nerve terminals,^84^ which might explain why its dysfunction may result in selective impairment of inhibitory transmission and the resulting electro‐clinical syndrome.

No distinct phenotypes are recognized for each serogroup, and the few neurophysiological studies published show the same pattern of CMUA.^9^, ^21^, ^55^ Moreover, the clinical improvement with centrally acting depressor drugs (GABA mimetic), irrespective of the serogroup, suggests a common mechanism underlying impaired GABAergic function^12^, ^85^; this notion is further supported by the similar clinical response and electrophysiological findings in seronegative SPSD patients.^86^ Nevertheless, it is important to consider that GABAergic drugs are effective in different conditions (including spasticity and dystonia), and their mechanisms of action could be nonspecific and not indicative of the underlying pathophysiology. Further studies are warranted to confirm the role of GABA in SPSD, but other potential therapeutic targets should be considered. By elucidating the abnormalities in neural inhibition and excitability, electrophysiologic studies might also open new therapeutic avenues in SPSD. A comprehensive neurophysiological evaluation addressing the multiple levels at which GABAergic dysfunction appears to be present might unravel upstream pathways to be targeted by centrally acting GABA mimetics or neuromodulation strategies. Other pathways mediated by related neurotransmitters (eg, glycine) may be additionally explored by further studies, with the potential to design specific treatment approaches. Moreover, electrophysiological biomarkers may help monitor disease progression and treatment response, facilitating personalized therapeutic approaches.

We found some limitations in the electrophysiological studies on SPSD published so far. Some of the reported findings, for instance, exaggerated startle responses, are not specific to SPSD, being present in other pathologic conditions.^87^, ^88^, ^89^ However, it is the combination of electrophysiological findings from different tested modalities pointing to hyperexcitability that is most characteristic of SPSD. Moreover, the protocol used to evaluate the same phenomenon (eg, the muscles used to assess CMUA or reflex responses) varies between different studies, thus limiting direct comparisons. Another issue concerns sample sizes, as most findings were obtained from case reports or relatively small series. This reflects the overall low prevalence of SPSD. Multicentric collaborations in which the same standardized protocol is applied would be helpful in overcoming these issues.

In conclusion, neurophysiology provides valuable insights into the pathophysiology of SPSD. A predominant inhibitory dysfunction across the CNS seems to be the defining characteristic of SPSD. Nevertheless, it remains uncertain if neurophysiological features mirror the diverse immune specificities within the spectrum of SPSD. Moving forward, a comprehensive electrophysiological assessment of spinal and supraspinal circuits in various SPSD serogroups could aid in resolving this uncertainty.

## Author Roles

(1) Research project: A. Conception, B. Organization, C. Execution; (2) Statistical analysis: A. Design, B. Execution, C. Review and critique; (3) Manuscript preparation: A. Writing of the first draft, B. Review and critique.

J.M.: 1A, 1B, 1C, 3A

L.R.: 1C, 3B

M.Z.: 3B

B.B.: 3B

K.P.B.: 3B

A.L.: 1A, 1B, 1C, 3A

## Disclosures

**Ethical Compliance Statement:** We confirm that the approval of an institutional review board was not required for this work. Informed patient consent was not necessary for this work. We confirm that we have read the journal's position on issues involved in ethical publication and affirm that this work is consistent with those guidelines.

**Funding Sources and Conflicts of Interest:** No specific funding was received for this work. The authors declare that there are no conflicts of interest relevant to this work.

**Financial Disclosures for the Previous 12 Months:** J.M. received a PhD research scholarship from ICBAS/BIAL Foundation and support to attend scientific conferences/meetings from Orphalan, Sanofi, Novartis, UCB, Alnylam, and Alexion. A.L. is supported by EPSRC and MRC under the NEUROMOD+ Network (EP/W035057/1). She received honoraria from the Movement Disorder Society for educational activities.

## Supporting information


**Table S1.** Summary of the included studies according to the proposed diagnostic criteria (Chia et al. 2023).

## Data Availability

Data sharing is not applicable to this article as no new data were created or analyzed in this study.
