# Supplementary material for: Neurophysiological Insights into the Pathophysiology of Stiff‐Person Spectrum Disorders
Source: Mov Disord Clin Pract. 2025 Jan 8;12(4):409–17. doi: 10.1002/mdc3.14328 (PMC11998688; doi:10.1002/mdc3.14328)
Supplement: Supplementary file 1 — Table S1. Summary of the included studies according to the proposed diagnostic criteria (Chia et al. 2023). [file MDC3-12-409-s001.docx]

Supplementary table 1: summary of the included studies according to the proposed diagnostic criteria (Chia et al. 2023)

| **Article** | **n** | **Clinical symptoms (stiffness, episodic spasms)** | **Clinical signs (increased tone, exaggerated lumbar lordosis, concurrent stiffness of lumbar paraspinal and abdominal muscles)** | **Serologic findings (high- titer GAD65 IgG in serum or any positive CSF titer; Glycine IgG in serum and/or CSF; Amphiphysine IgG in serum and/or CSF)** | **Electrophysiological studies (persistent activity in needle EMG; exaggerated reflex responsed by surface EMG; co-contraction of agonist/antagonist)** |
| --- | --- | --- | --- | --- | --- |
| Barker et al. (1998)^5^ | 23 | Yes | Yes | Yes in 9/23 | Yes |
| Raju et al. (2006)^8^ | 25 | Yes | Yes | Yes | Yes |
| Murinson et al. (2008)^9^ | 127 | Yes | Detailed in 94 | 116 GAD  11 Amphiphysine | Yes in 80% |
| Meinck et al. (1995)^11^  Meinck et al. (1993) ^14^ | 8 | Yes | Yes | 4 GAD | Yes |
| Martinelli et al. (1978)^16^ | 1 | Yes | Yes | No reported | Yes |
| Mamoli et al. (1977)^17^ | 1 | Yes | Yes | Not reported | Yes |
| Saiz et al. (1998)^19^ | 2 | Yes | Yes | Yes | Yes |
| Picciolo et al. (2001)^20^ | 1 | Yes | Yes | Yes* | Yes |
| Maeda et al. (2019)^21^ | 1 | Yes | Yes | Yes* | Yes |
| Lorenzoni et al. (2012)^22^ | 4 | Yes | Yes | Yes* | Yes (CMUA in ¾; co-contraction in 2/4). |
| Ehler et al. (2011)^23^ | 1 | Yes | Yes | Yes | Yes |
| Chang et al. (2016)^24^ | 1 | Yes | Yes | Yes | Yes |
| Meinck et al. (1984)^29^ | 1 | Yes | Yes | No | Yes |
| Floeter et al. (1998)^35^ | 11 | Yes | Yes | Yes* | Yes in 8/11 (CMUA in 8/11; co-contraction in 6/8) |
| Martinelli et al. (1996)^36^ | 1 | Yes | Yes | Yes* | Yes |
| Nalbantoglu et al. (2016)^37^ | 1 | Yes | Yes | Yes | Yes |
| Matsumoto et al. (1994)^40^ | 8 | Yes | Yes | Yes* | Yes |
| Brashear et al. (1991)^41^ | 1 | Yes | Yes | Yes | Yes |
| Kullmann et al. (1996)^43^ | 2 | Yes | Yes | No | Yes |
| McKeon et al. (2012)^44^ | 99 | Yes | Yes | 79/99 GAD; 3/99 amphiphysine | 28/51 with abnormal results |
| Khasani et al. (2004)^45^ | 10 | Yes | Yes | 8/10 GAD | 4/10 with altered reflex responses |
| Berger et al. (2003)^48^ | 30 | Yes | Yes | 17/30 GAD | 17/30 altered head retraction reflex |
| Molloy et al. (2002)^54^ | 4 | Yes | Yes | No | Yes (altered reflex responses) |
| Irie et al. (2022)^55^ | 1 | Yes | Yes | Yes | Yes |
| Sandbrink et al. (2000)^62^ | 7 | Yes | Yes | Yes | Yes |
| Logullo et al. (1999)^65^ | 1 | Yes | Yes | Yes | Yes |
| Koerner et al. (2004)^66^ | 21 | Yes | Yes | 15 | Yes |
| Rossi et al. (2010)^69^ | 1 | Yes | Yes | Yes | TMS parameters reported |

* - antibody positive, no titer reported;
